# Adverse events associated with medical cannabis reported within a centralized call center

**DOI:** 10.3389/fphar.2026.1792520

**Published:** 2026-05-04

**Authors:** Sílvia M. Illamola, Xintian Lyu, Monica Luciana, Stephen Dahmer, Paloma Lehfeldt, Sibel Dikmen, Inonge Kaloustian, Marne Garretson, Richard L. Kingston, Ilo Leppik, Catherine M. Sherwin, Angela K. Birnbaum

**Affiliations:** 1 Department of Experimental and Clinical Pharmacology, College of Pharmacy, University of Minnesota, Minneapolis, MN, United States; 2 Department of Psychology, University of Minnesota, Minneapolis, MN, United States; 3 Andrew Weil Center for Integrative Medicine (AWCIM), Department of Family and Community Medicine, University of Arizona College of Medicine, Tucson, AZ, United States; 4 Goodness Growth Holdings, Minneapolis, MN, United States; 5 BCIT - British Columbia Institute of Technology: Adjunct Faculty, Burnaby, BC, Canada; 6 Vireo Health, Minneapolis, MN, United States; 7 Department of Epidemiology, Johns Hopkins Bloomberg School of Public Health, Baltimore, MD, United States; 8 SafetyCall International, LLC, Bloomington, MN, United States; 9 Center for Clinical and Cognitive Neuropharmacology, College of Pharmacy, University of Minnesota, Minneapolis, MN, United States; 10 Department of Neurology, School of Medicine, University of Minnesota, Minneapolis, MN, United States; 11 Department of Pharmacology and Toxicology, Wright State University Boonshoft School of Medicine, Dayton, OH, United States; 12 Internal Medicine, UWA Medical School, The University of Western Australia, Perth, WA, Australia; 13 Department of Medical Laboratory Sciences, College of Pharmacy, University of Minnesota, Minneapolis, MN, United States

**Keywords:** adverse events, cannabinoids, medical cannabis, neuropharmacology, pharmacovigilance, real-world evidence, delta-9-tetrahydrocannabinol

## Abstract

**Introduction:**

The use of medical cannabis products is expanding, yet real-world data on associated adverse events (AEs) remain limited. Controlled trials often exclude diverse patient populations and product types, making post-marketing surveillance essential to understanding cannabinoid safety.

**Objective:**

The aim of this study is to characterize AEs reported by patients enrolled in the Minnesota Medical Cannabis Program and explore associations between AE severity, cannabinoid doses, and product types.

**Materials and methods:**

This was a retrospective analysis of AEs reported between 2015 and 2021 by individuals receiving products from a single licensed manufacturer. Demographic data, qualifying condition, cannabis formulation, and daily purified cannabidiol (CBD)/delta-9-tetrahydrocannabinol (THC) doses were collected. AEs were classified by severity and analyzed in relation to cannabinoid content and product formulation using non-parametric Mann–Whitney U tests (*p* < 0.05).

**Results:**

A total of 237 calls were received from 225 individuals reporting 692 symptoms. Most calls were from medical cannabis consumers (79.3%) and were predominantly classified as minor in severity (71.7%). Most AEs were associated with THC-dominant products (39.8%), and capsule formulations (36.8% of the overall products) were most commonly implicated. Among individuals with dose data, those with moderate AEs were associated with significantly higher daily THC doses than those with minor AEs (*p* < 0.05). Treatment discontinuation occurred in 32.5% of cases following AE reporting.

**Conclusion:**

Although AEs were infrequently reported, they were often clinically meaningful and led to treatment discontinuation. The voluntary nature of reporting likely underestimates the actual AE burden, capturing only more severe or bothersome events. These findings underscore the need for enhanced pharmacovigilance systems and further research into the long-term safety and public health implications of cannabinoid therapies, especially among medically complex patients.

## 1 Introduction

The general and medical use of cannabis products in adults is expanding rapidly as many states legalize their use due to changes in public sentiment. The cannabis plant has a rich profile of approximately 100 biologically active constituents, each found in varying amounts. The complexity of this rich profile is further compounded by the wide variety of cannabis products currently available in the United States (Andre et al., 2016). FDA-approved products contain a single agent, either purified cannabidiol (CBD) (Greenwich Biosciences Inc Epidiolex, 2021) or dronabinol (synthetic delta-9-tetrahydrocannabinol: THC) (Benuvia Therapeutics Inc Syndros, 2020; Patheon Softgels Inc Marinol, 2017). They are indicated for specific patient populations with particular medical conditions and offered in restricted and specific formulations.

The pharmacokinetics of CBD and THC are complex, with substantial inter-individual variability in drug exposure and physiological responses. Pharmacokinetic variability is attributed to several factors, including high lipophilicity (Chayasirisobhon, 2020; Gaston and Friedman, 2017; Agurell et al., 1981), significant food effects on drug absorption (Birnbaum et al., 2019; Mozaffari et al., 2021), bioavailability based on delivery forms (Chayasirisobhon, 2020; Lucas et al., 2018), and significant first-pass metabolism (Eichler et al., 2012; Schwope et al., 2011; Zendulka et al., 2016), resulting in potential drug–drug interactions with prescribed or over-the-counter medications. CBD and THC are primarily metabolized by cytochrome P450 (CYP) enzymes, including CYP2C9, CYP2D6, CYP2C19, and CYP3A4. Both cannabinoids have been shown to inhibit CYP2C9, CYP2D6, and CYP2C19 *in vitro* with various degrees of potency (Zendulka et al., 2016). Cannabis is frequently used concomitantly with psychotropic medications, including antidepressants and anxiolytics (Bahorik et al., 2017). Several selective serotonin reuptake inhibitors (SSRIs), such as citalopram and sertraline, are primarily metabolized by CYP2C19, and escitalopram is metabolized by both CYP2C19 and CYP2D6 (Hicks et al., 2015; Brown and Winterstein, 2019). Therefore, inhibition of P450 enzymes by CBD, THC, or both may lead to clinically relevant drug–drug interactions. In addition, CBD has been shown to inhibit P-glycoprotein activity *in vitro* (Zhu et al., 2006). Some SSRIs are substrates of P-glycoprotein at the blood–brain barrier, which suggests a potential transporter-mediated contribution to altered central nervous system exposure (O'Brien et al., 2012; O'Brien et al., 2013). Furthermore, data indicate that concomitant use of medical cannabis and certain SSRIs may increase the risk of unintended adverse events (Chrobak et al., 2024; Vaughn et al., 2021).

THC is the primary psychoactive constituent of cannabis and acts as a partial agonist at both CB1 and CB2 receptors, exhibiting high affinity for these sites (Iwamura et al., 2001; Rinaldi-Carmona et al., 1994; Bayewitch et al., 1996; Showalter et al., 1996; Rhee et al., 1997). Activation of CB1 receptors mediates the inhibition of the ongoing release of multiple excitatory and inhibitory neurotransmitters [e.g., acetylcholine, noradrenaline, glutamate, and γ-aminobutyric acid (GABA)] (Pertwee, 2008), with effects in brain regions enriched with CB1 receptors, including the hippocampus, prefrontal cortex, amygdala, and cerebellum (Mackie, 2005). In contrast, CBD demonstrates low affinity for CB1 and CB2 receptors and acts as a high-potency antagonist (Showalter et al., 1996; MacLennan et al., 1998; Bisogno et al., 2001; Pertwee, 2008). CBD has shown potential in ameliorating or counteracting some of the adverse effects often associated with THC (MacCallum and Russo, 2018). However, a study reported that co-administration of THC with high doses of CBD may increase the probability of adverse events (AEs) compared to a similar dose of THC without CBD as CBD inhibits the metabolism of THC (Zamarripa et al., 2023; Jones and Pertwee, 1972). Several clinical trials involving selected patient groups have been conducted to assess the safety of CBD and THC alone (de Carvalho Reis et al., 2020; Devinsky et al., 2017; Chesney et al., 2020; Huestis et al., 2019; Ware et al., 2015; Almog et al., 2020; Kayser et al., 2020). These trials have included limited dosage regimens, specific formulations, highly restricted patient populations, and particular queries about AEs. As a result, a significant knowledge gap persists in understanding the real-world AEs associated with medical consumption of THC and CBD, particularly with the more diverse array of doses, formulations, and users. In this study, we aimed to take advantage of the reporting system in Minnesota to characterize self-reported AEs occurring through a centralized surveillance system. We also sought to correlate reported AEs with specific doses and product types to identify the AEs of most concern to users.

## 2 Materials and methods

### 2.1 Data source and patient population

We use retrospective data entered into a centralized database (SafetyCall, Bloomington, MN) for patients consuming cannabis products from a single medical cannabis (MC) manufacturer (Vireo Health). Participants in the Minnesota state program must read and sign a Tennessen Notice and Acknowledgement form. State statute also specifies the use of patient data for scientific, peer-reviewed publication of research (Minnesota Statute 152.28). The University of Minnesota Human Subjects Committee reviewed the study, and a material transfer agreement was signed between Vireo Health and the University of Minnesota.

The Minnesota (MN) Medical Cannabis program, launched on 1 July 2015, provides dosing supervision by pharmacists in collaboration with patients participating in the program. Program participants must be certified for qualifying conditions by a Minnesota-licensed practitioner, registered with the state, and present themselves at a dispensary where specific formulations are recommended. At the time of this study, there were 17 qualifying conditions: cancer associated with severe/chronic pain, nausea or vomiting, or cachexia or severe wasting; glaucoma; HIV/AIDS; Tourette syndrome; amyotrophic lateral sclerosis; seizures, including those characteristic of epilepsy; severe and persistent muscle spasms, including those characteristic of multiple sclerosis; inflammatory bowel disease, including Crohn’s disease; terminal illness, with a probable life expectancy of <1 year; intractable pain; posttraumatic stress disorder; autism; obstructive sleep apnea; Alzheimer disease; chronic pain; sickle cell disease; and chronic motor or vocal tic disorder (Minnesota Department of Health Office of Medical Cannabis, 2023). Persons receiving cannabis products under the Minnesota program are requested to report AEs to SafetyCall International, a third-party internationally recognized poison control and AE call center that operates on a 24/7 basis. Toxicology- and pharmacovigilance-trained healthcare professional staff members answer each call and investigate, manage, and document reported AEs associated with the use of MC products. AEs are reported directly from consumers or others (e.g., family members and healthcare professionals). Information collected regarding each reported AE is aggregated in a Title 21 CFR Part 11-compliant database.

### 2.2 Data collection and analysis

We combined two separate retrospective datasets from a single MC manufacturer for this analysis through unique identifiers available in both datasets. The first dataset was data reported to SafetyCall (1 July 2015 to 12 September 2021) and included AEs reported from patients consuming cannabis products from Vireo Health, representing 17 qualifying conditions available in 2021. Recognized AEs included any health-related effect associated with product use (e.g., cough, diarrhea, or mental status change). Information not recognized as an AE (i.e., expired product dispensed, efficacy issue, device breakage, condition aggravated, or overdose) was excluded from the analysis. Cases classified as “condition aggravated” were excluded because worsening of the underlying disease could not be reliably attributed to cannabis exposure and may instead reflect natural disease progression. Reports categorized as “overdose” were also excluded as they represent use outside recommended dosing and therefore were not considered adverse events occurring under typical therapeutic conditions. The second dataset provided dispensary-level information regarding the qualifying condition (diagnosis) for recommending MC, dispensary visit dates, dispensed products, and dosage information. The dispensary dataset included the total number of individuals dispensed an MC product from 2016 to 2020. In seven cases, the first dispensary date was unavailable because it occurred before dispensary data collection began (16 June 2016) and was therefore counted as missing data. Thus, the data were excluded from this analysis.

Data provided to SafetyCall document AEs using a standard-of-care adverse event data collection tool. Data collected during calls included caller, site of exposure (i.e., patient residence or workplace), patient demographics (i.e., gender, age, and weight), medical information of the patient (e.g., smoker status, reported allergies, and prior medical history), information about MC products used (e.g., name, route, formulation, and dose), outcome characteristics (e.g., symptoms, duration, and severity), and a whole narrative describing the incident and reported AE(s).

Individuals could report one or multiple signs/symptoms (s/s) related to MC consumption. Undefined s/s included cases when the individual reported an AE but could not provide details (e.g., unspecified s/s, less functional, or unspecified type). Information on AEs was reported by severity (i.e., asymptomatic–no effect, minor, moderate, major, and death), following the data collection and reporting format of the American Association or Poison Control Centers. Reported events were defined as asymptomatic (no effect) when individuals did not present AEs. Events were defined as “major” when the reported cases resulted in hospitalization, prolongation of hospitalization, or persistent or significant disability/incapacity.

In our study, events were further classified according to the organ system based on the International Classification of Diseases and Related Health Problems (ICD-10). The ICD-10 is a standardized system for classifying medical diagnoses and procedures, developed by the World Health Organization (World Health Organization, 2025; ICD10DATA, 2022). Central nervous system-related adverse events included R40–46 (symptoms and signs involving cognition, perception, emotional state, and behavior), F (mental, behavioral, and neurodevelopmental disorders), and G (diseases of the nervous system). MC product(s) responsible for the reported events were classified as follows: (1) “CBD only,” (2) “CBD dominant” (low THC levels and high CBD levels), (3) “balanced THC/CBD” (comparable levels of THC and CBD), (4) “THC only,” (5) “THC-dominant” (low CBD levels), and (6) “starter pack” (product trial of formulations). The “starter pack” contained MC products with different THC to CBD compositions and was usually used to initiate the treatment.

The data were further stratified by the number of reported products: (1) single product indicated as responsible for the AEs; (2) multiple MC products were prescribed; and (3) MC product not reported. For the single-product category, we additionally explored the relationship between different characteristics of the MC products used (i.e., composition, formulation, and dose) and the severity of the reported events. We compared doses between different severity groups using non-parametric Mann–Whitney U tests (p < 0.05). Data analysis was performed using R version 4.0.3.

## 3 Results

### 3.1 Calls reported regardless of the number of cannabis products

Information from 241 calls (229 unique callers) reporting AEs from MC products was available. Data for 2015, 2016, and 2021 were not included due to the incompleteness of the time-matched dispensary data. Four calls that did not directly report AEs (e.g., expired product dispensed) were excluded, resulting in 237 calls (225 unique individuals) reporting 692 s/s. Patients were primarily female (59.5%) with a median age of 60 years.

The median number of s/s reported in each call was 2 (range: 1–12), with intractable pain (40.1%) being the most frequent qualifying condition (Supplementary Table S1). Most calls were from MC consumers (79.3%), followed by healthcare providers (11.8%), mainly pharmacists (10.5%). The number of major and moderate AEs was higher when consumers reported the events (Table 1). In five cases, a second follow-up call was conducted. The person reporting the event (e.g., self-reported and healthcare provider) was similar across years (data not shown).

**TABLE 1:** Number and percentage of calls by the role of the initial caller reporting the adverse events.

Severity				
Role	Minor	Moderate	Major	Total
Self	139 (74.0%)	45 (23.9%)	2 (1.1%)	188 (79.3%)
Pharmacist	15 (60.0%)	10 (40.0%)	0 (0.0%)	25 (10.5%)
Family	9 (75.0%)	3 (25.0%)	0 (0.0%)	12 (5.1%)
Other	3 (60.0%)	2 (40.0%)	0 (0.0%)	5 (2.1%)
Unknown	2 (50.0%)	2 (50.0%)	0 (0.0%)	4 (1.7%)
Physician	1 (50.0%)	1 (50.0%)	0 (0.0%)	2 (0.8%)
Other healthcare professionals	1 (100.0%)	0 (0.0%)	0 (0.0%)	1 (0.4%)

*Two self-reported calls were classified as asymptomatic and unknown, respectively.

Among the 237 calls, 71.7% reported minor, 26.6% reported moderate, and 0.8% (2 calls) reported major s/s. Two calls could not be classified based on severity: one reported the event as asymptomatic, and another call reported it as “undefined.” The reported major s/s included cough, dyspnea/shortness of breath, respiratory irritation, worsened anxiety, chills/rigors, dizziness/vertigo, drowsiness/lethargy, emesis/vomiting, feeling high, fever/hyperthermia, insomnia/sleep disorder, lung infection, and nausea. Among individuals <18 years old (n = 11; 4.6%), reported s/s were evenly distributed between minor (54.5%) and moderate (45.5%) (Table 2). The severity of the reported s/s was similar across years (data not shown). S/s classified as “ICD-10 R” were the most frequently reported (72.3%) (Supplementary Table S2), specifically “ICD-10 R40–46” (32.4%) (e.g., anxiety and agitated/irritable), “ICD-10 R10–19” (20.4%) (e.g., vomiting and diarrhea), and “ICD-10 R50–69” (19.0%) (e.g., increased appetite and weight loss). The most common specific s/s were cognitive (18.9%), nausea and vomiting (17.8%), and dizziness and giddiness (11.7%) (Table 3).

**TABLE 2:** Number and percentage of calls by symptom severity, age, and gender.

Severity					
Sub-group	Category	Minor (N = 170)	Moderate (N = 63)	Major (N = 2)	Total (N = 237)
Age	<18	6 (54.5%)	5 (45.5%)	0 (0.0%)	11 (4.6%)
	18–64	84 (70.0%)	33 (27.5%)	2 (1.7%)	120 (50.6%)
	≥65	71 (76.3%)	22 (23.7%)	0 (0.0%)	93 (39.2%)
	Unknown	9 (69.2%)	3 (23.1%)	0 (0.0%)	13 (5.5%)
Gender	Female	105 (74.5%)	33 (23.4%)	1 (0.7%)	141 (59.5%)
	Male	62 (67.4%)	29 (31.5%)	1 (1.1%)	92 (38.8%)
	Unknown	3 (75.0%)	1 (25.0%)	0 (0.0%)	4 (1.7%)

*One call was asymptomatic and involved a female patient of unknown age. Another call was of unknown severity and involved a female patient aged 18–64 years.

**TABLE 3:** Most reported symptoms within ICD-10 R40–46, ICD-10 R10–19, and ICD-10 R50–69 groups.

	Severity			
Symptoms by ICD-10 codes	Minor	Moderate	Major	Total
Other symptoms and signs involving cognitive functions and awareness	43 (63.2%)	23 (33.8%)	2 (2.9%)	68 (18.9%)
Nausea and vomiting	36 (56.3%)	26 (40.6%)	2 (3.1%)	64 (17.8%)
Dizziness and giddiness	33 (78.6%)	8 (19.0%)	1 (2.4%)	42 (11.7%)
Symptoms and signs involving emotional state	20 (71.4%)	8 (28.6%)	0 (0.0%)	28 (7.8%)
Headache	18 (81.8%)	4 (18.2%)	0 (0.0%)	22 (6.1%)
Malaise and fatigue	12 (54.5%)	10 (45.5%)	0 (0.0%)	22 (6.1%)
Other symptoms and signs involving the digestive system and abdomen	14 (77.8%)	4 (22.2%)	0 (0.0%)	18 (5.0%)
Pain, unspecified	11 (73.3%)	4 (26.7%)	0 (0.0%)	15 (4.2%)
Somnolence, stupor, and coma	9 (69.2%)	3 (23.1%)	1 (7.7%)	13 (3.6%)
Abdominal and pelvic pain	10 (83.3%)	2 (16.7%)	0 (0.0%)	12 (3.3%)
Other general symptoms and signs	9 (75.0%)	2 (16.7%)	1 (8.3%)	12 (3.3%)
Symptoms and signs concerning food and fluid intake	8 (72.7%)	3 (27.3%)	0 (0.0%)	11 (3.1%)
Other symptoms and signs involving general sensations and perceptions	6 (60.0%)	4 (40.0%)	0 (0.0%)	10 (2.8%)
Syncope and collapse	1 (20.0%)	4 (80.0%)	0 (0.0%)	5 (1.4%)
Flatulence and related conditions	4 (100.0%)	0 (0.0%)	0 (0.0%)	4 (1.1%)
Aphagia and dysphagia	2 (66.7%)	1 (33.3%)	0 (0.0%)	3 (0.8%)
Fever of other and unknown origin	0 (0.0%)	2 (66.7%)	1 (33.3%)	3 (0.8%)
Generalized hyperhidrosis	2 (66.7%)	1 (33.3%)	0 (0.0%)	3 (0.8%)
Edema, not elsewhere classified	0 (0.0%)	2 (100.0%)	0 (0.0%)	2 (0.6%)
Disturbances of smell and taste	1 (100.0%)	0 (0.0%)	0 (0.0%)	1 (0.3%)
Heartburn	0 (0.0%)	1 (100.0%)	0 (0.0%)	1 (0.3%)

Dispensary information on the MC products was available in 67.5% of the calls (n = 160). The events reported from these 160 calls occurred from 0 to 39 months (median, 15 months) after the first dispensation. From these 160 calls, 52 cases (32.5%) did not have a future dispensary visit logged after reporting the AEs, indicating that the treatment was discontinued. The percentage of calls reporting a single or multiple cannabis products responsible for the AEs was 56.1% and 40.9%, respectively. Only 3.0% of calls did not report the MC product accountable for the AEs.

### 3.2 Calls reporting only one medical cannabis product

**FIGURE 1:**
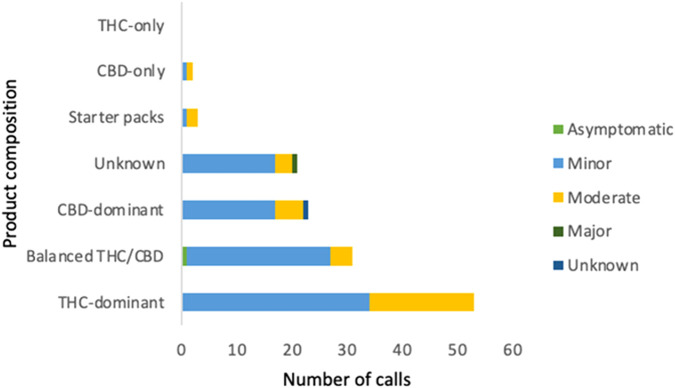
Number of calls stratified by product composition and severity of reported adverse events.

For the group reporting only one MC product (N = 133 calls), s/s mainly were associated with “THC-dominant” products (N = 53 calls), followed by “balanced THC/CBD” products (N = 31 calls) (Figure 1). Starter packs were considered a single product and reported in three calls (two moderate and one minor s/s). By formulation, s/s were primarily related to capsules (36.8%), except in 2021 (Table 4), followed by vaporizers (16.5%) and tinctures (11.3%).

**TABLE 4:** Number and percentage of calls by symptom severity and formulation.

​	Severity			
Product	Minor (N = 96)	Moderate (N = 34)	Major (N = 1)	Total (N = 133)
Capsule	37 (75.5%)	12 (24.5%)	0 (0.0%)	49 (36.8%)
Vaporizer	14 (63.6%)	7 (31.8%)	1 (4.5%)	22 (16.5%)
Tincture	11 (73.3%)	3 (20.0%)	0 (0.0%)	15 (11.3%)
Oral solution	9 (90.0%)	1 (10.0%)	0 (0.0%)	10 (7.5%)
Bulk oil	2 (25.0%)	6 (75.0%)	0 (0.0%)	8 (6.0%)
Topical	2 (50.0%)	2 (50.0%)	0 (0.0%)	4 (3.0%)
Tablets	2 (100.0%)	0 (0.0%)	0 (0.0%)	2 (1.5%)
Oral spray	0 (0.0%)	0 (0.0%)	0 (0.0%)	1 (0.8%)
Unknown	19 (86.4%)	3 (13.6%)	0 (0.0%)	22 (16.5%)

*One call was asymptomatic (tincture), and another had unknown severity (oral spray).

Stratified by severity, minor s/s were the most frequent, regardless of product and formulation used, except for bulk oil, which had more moderate (75%) than minor s/s (25%), and for topical formulation, where minor and moderate s/s were equally reported (50%). Only eight and four cases were reported for bulk-oil and topical formulations, respectively. The only major s/s reported was related to the use of vaporizers, but the product composition used was not reported. When events were either asymptomatic or had missing severity information, they were associated with the use of tinctures and oral sprays, respectively (Table 4).

#### 3.2.1 Analysis of dose versus severity of adverse events

Among calls reporting a single MC product (N = 133), 33.1% (n = 44) had dose information available. The median (range) CBD and THC daily doses were 14.6 mg/day (1.3–215.9 mg/day) and 15.0 mg/day (2.5–1,000.0 mg/day), respectively. The daily dose for the only reported major adverse event was not available. The median (range) CBD daily dose stratified by adverse event severity was 10.4 (1.3–197.9) mg/day and 15.0 (4.3–215.9) mg/day for minor (n = 22) and moderate (n = 10) AEs, respectively. The median (range) THC daily dose stratified by AE severity was 10.7 (2.5–250.0) mg/day and 45.8 (2.5–1,000.0) mg/day for minor (n = 29) and moderate (n = 18) AEs, respectively. THC daily doses were significantly higher for moderate events than for minor events (*p* < 0.05). A total of 20.5% of individuals discontinued visits after reporting s/s (five minor and four moderate). The remaining 79.5% of individuals, who had subsequent visits after reporting s/s and continued with the same product associated with the reported AEs, did not show any pattern in dose change (data not shown).

## 4 Discussion

Although most AEs were classified as minor, one-third of persons contacting the call center subsequently discontinued use of cannabis products. Our study and previous reports (Vickery et al., 2022; MacNair et al., 2022; Erridge et al., 2022; Cahill et al., 2021) demonstrate the potential for AEs from the use of cannabis products. The severity of AEs is usually classified according to the need for medical intervention. Our study is unique in that we reported on AEs that were of sufficient concern, prompting reports to a centralized adverse event call center.

A variety of AEs are associated with CBD, including drowsiness, seizure worsening, and weight loss (de Carvalho Reis et al., 2020; Devinsky et al., 2017; Chesney et al., 2020; Huestis et al., 2019). Similarly, THC presents comparable AEs pertaining to a variety of organ systems, including the central nervous, gastrointestinal, and respiratory systems, along with those classified as general symptoms and signs (e.g., dry mouth and pain) (Ware et al., 2015; Almog et al., 2020). Our study showed a similar pattern, with AEs varying among users, likely due to differences in product constituents and pharmacokinetics across formulations. Bioavailability varies across individuals and formulations and is impacted by fasting or fed states (Lunn et al., 2019; Millar et al., 2018; Birnbaum et al., 2019; Hryhorowicz et al., 2018), thus introducing an increased potential for AEs. In our study, 35.5% of AEs were related to the central nervous system, which could be due to THC binding to the CB1 receptor.

Sex and genetic differences may play a role in how cannabinoids affect individuals (MacNair et al., 2024; Urits et al., 2021); however, genetic information in our dataset was limited. In addition, pre-existing physiological conditions may determine which AEs are most likely to be experienced. A person with underlying depression or anxiety may experience a different set of AEs than a person with a pain syndrome with no underlying issues. Of the two calls classified as severe, only one had a reported formulation. It was associated with vaping; therefore, its appearance could be due to pulmonary irritation rather than the particular cannabinoid. Both demographic information and the presence of comorbidities would improve the ability to assess the appearance of adverse events. However, qualifying condition information was missing in 32.5% of the cases (Supplementary Table S1).

Our study found 237 AEs reported over 6 years. Differences in the percentage of AEs in our study compared to others (11.7%–32.3%) may be attributed to the voluntary reporting design of the Minnesota program (Vickery et al., 2022; MacNair et al., 2022; Erridge et al., 2022), which differs from studies that include a specific follow-up protocol and questionnaires to identify related adverse events. Increased AEs may also result from drug–drug interactions in specific population differences. In a meta-analysis of placebo-controlled studies of CBD, a high rate of AEs leading to discontinuation was observed only in the pediatric population with epilepsy who were treated with concomitant anti-seizure medications (Chesney et al., 2020). In the Minnesota program, patients are encouraged to report AEs (by regulations, providers, pharmacists, and labels on MC products). The reported signs and symptoms were of significant concern, and the users and caregivers were motivated to call the adverse event center. Other studies used data extracted from registries based on clinical visits where clinicians actively elicited potential AEs associated with an individual’s treatment. As our research relies on voluntary reporting of AEs to an international center, individuals in our study perceived AEs as significant enough to motivate a call to the center and a potential reason for discontinuation. In the Minnesota program, users are responsible for the full cost of the products, which introduces financial constraints as a possible reason for discontinuation of the product.

Unique to this AE reporting system is the fact that cannabis patients agree and are required by the medical cannabis program to report any AEs they experience as a condition of enrollment. Each call registered multiple symptoms experienced by the same individual, with a unique and overall severity description. Therefore, specifying the severity for each sign and symptom was impossible. In addition, the dataset used in the analysis included product and AE information exclusively from a single licensed manufacturer, which is the only available source of structured, linkable AE and dispensary data within the Minnesota Medical Cannabis Program during the study period. As such, it was not possible to compare across multiple brands or evaluate brand-specific differences in AE rates. Starter packs were introduced at the beginning of medical cannabis treatment to facilitate product selection and potentially reduce adverse events during treatment. We decided to include starter packs in our analysis as this reflects clinical practice, and the exclusion of starter packs from the analysis could underestimate the incidence of adverse events and potentially bias the results.

Naturalistic methodology aims to observe AEs as they would occur in an individual’s living environment, with very little study design interference. Considering that the vast majority of cannabis consumers in the US do not consume an FDA-approved product and are unlikely to do so shortly, more observations from individuals in state programs can be helpful. Our study and others (Vickery et al., 2022; MacNair et al., 2022; Erridge et al., 2022; Cahill et al., 2021) show that there is a large spectrum of AEs associated with THC and CBD. The individual AE experience is based primarily on factors such as physiopathological factors, including the user’s physiology, concomitant disorder(s), and psychiatric state. The specific delivery form (i.e., gummy, cookie, etc.) must also be considered. Finally, the dose of THC and CBD may be inaccurate when reported by patients due to a lack of recall or variability in bioavailability across products and formulations. Concerning trends in emergency room visits for adverse reactions to cannabis products already exist (Varin et al., 2023). Appropriate documentation, diagnosis, and treatment must be based on an improved understanding of AEs specific to an individual’s clinical circumstances.

## 5 Conclusion

Although the number of AEs related to the use of medical cannabis was low in this study, when present, they could be significant enough to discontinue treatment. AEs captured in this study were those of sufficient concern to prompt patients or caregivers to contact a centralized call center, suggesting that only more severe or bothersome AEs were reported. Notably, the THC daily dose was higher in more severe AEs. This study highlights the need for enhanced pharmacovigilance and structured surveillance systems to better quantify and characterize cannabinoid-associated AEs in real-world settings. Further research should prioritize understanding the long-term safety and public health implications of cannabinoid use, particularly in populations with comorbidities, polypharmacy, or prolonged exposure.

## Data Availability

The datasets generated and analyzed during the current study are not publicly available due to patient confidentiality and state data use restrictions but are available from the corresponding author on reasonable request and with appropriate institutional and regulatory approvals. Aggregated summary data supporting the findings of this study are included within the manuscript and supplementary materials.
