# Supplementary material for: Adverse events associated with medical cannabis reported within a centralized call center
Source: Front Pharmacol. 2026 May 4;17:1792520. doi: 10.3389/fphar.2026.1792520 (PMC13181341; doi:10.3389/fphar.2026.1792520)
Supplement: Supplementary file 1 [file Table1.docx]

**Supplement Table 1. Percentage of qualifying conditions associated with the use of medical cannabis**

|  | **Severity** | | | |
| --- | --- | --- | --- | --- |
| **Condition** | **Minor** | **Moderate** | **Major** | **Total** |
| ALS | 1 (100.0%) | 0 (0.0%) | 0 (0.0%) | 1 (0.4%) |
| Cancer | 6 (100.0%) | 0 (0.0%) | 0 (0.0%) | 6 (2.5%) |
| Chronic pain | 5 (83.3%) | 1 (16.7%) | 0 (0.0%) | 6 (2.5%) |
| Intractable pain | 67 (70.5%) | 26 (27.4%) | 0 (0.0%) | 95 (40.1%) |
| MS | 5 (83.3%) | 1 (16.7%) | 0 (0.0%) | 6 (2.5%) |
| Multiple Conditions | 15 (68.2%) | 6 (27.3%) | 1 (4.5%) | 22 (9.3%) |
| PTSD | 6 (54.5%) | 4 (36.4%) | 1 (9.1%) | 11 (4.6%) |
| Seizures | 4 (33.3%) | 8 (66.7%) | 0 (0.0%) | 12 (5.1%) |
| Terminal illness | 1 (100.0%) | 0 (0.0%) | 0 (0.0%) | 1 (0.4%) |
| NA | 60 (77.9%) | 17 (22.1%) | 0 (0.0%) | 77 (32.5%) |
| Two calls with intractable pain reported  asymptomatic and unknown-severity adverse events.  *QUALIFYING CONDITIONS during the study time period: (1) cancer associated with severe/chronic pain, nausea or vomiting, or cachexia or severe wasting; (2) glaucoma; (3) HIV/AIDS; (4) Tourette syndrome; (5) amyotrophic lateral sclerosis (ALS); (6) seizures, including those characteristic of epilepsy; (7) severe and persistent muscle spasms, including those characteristic of multiple sclerosis (MS); (8) inflammatory bowel disease, including Crohn disease; (9) terminal illness, with a probable life expectancy of < 1 year;(10) intractable pain; (11) posttraumatic stress disorder (PTSD); (12) autism; (13) obstructive sleep apnea; (14) Alzheimer disease; (15) chronic pain; (16) sickle cell disease; (17) chronic motor or vocal tic disorder.* | | | | |
