# Supplementary material for: Adverse events associated with medical cannabis reported within a centralized call center
Source: Front Pharmacol. 2026 May 4;17:1792520. doi: 10.3389/fphar.2026.1792520 (PMC13181341; doi:10.3389/fphar.2026.1792520)
Supplement: Supplementary file 2 [file Table2.docx]

**Supplement Table 2. Reported adverse events (signs/symptoms) by organ system classification**

|  |  | Severity |  |  |  |
| --- | --- | --- | --- | --- | --- |
| Organ system | **Minor** | **Moderate** | **Major** | **Unknown** | **Total** |
| Symptoms, signs and abnormal clinical and laboratory findings, not elsewhere classified (R) | 334 (66.8%) | 156 (31.2%) | 10 (2.0%) | 0 (0.0%) | 500 (72.3%) |
| Mental, Behavioral and Neurodevelopmental disorders (F) | 33 (63.5%) | 16 (30.8%) | 0 (0.0%) | 3 (5.8%) | 52 (7.5%) |
| Diseases of the nervous system (G) | 16 (50.0%) | 15 (46.9%) | 1 (3.1%) | 0 (0.0%) | 32 (4.6%) |
| Diseases of the skin and subcutaneous tissue (L) | 11 (36.7%) | 19 (63.3%) | 0 (0.0%) | 0 (0.0%) | 30 (4.3%) |
| Diseases of the ear and mastoid process (H) | 12 (60.0%) | 8 (40.0%) | 0 (0.0%) | 0 (0.0%) | 20 (2.9%) |
| Diseases of the digestive system (K) | 11 (73.3%) | 4 (26.7%) | 0 (0.0%) | 0 (0.0%) | 15 (2.2%) |
| Diseases of the musculoskeletal system and connective tissue (M) | 9 (69.2%) | 4 (30.8%) | 0 (0.0%) | 0 (0.0%) | 13 (1.9%) |
| Unclassified | 8 (72.7%) | 3 (27.3%) | 0 (0.0%) | 0 (0.0%) | 11 (1.6%) |
| Diseases of the circulatory system (I) | 1 (14.3%) | 6 (85.7%) | 0 (0.0%) | 0 (0.0%) | 7 (1.0%) |
| Diseases of the respiratory system (J) | 1 (20.0%) | 2 (40.0%) | 2 (40.0%) | 0 (0.0%) | 5 (0.7%) |
| Endocrine, nutritional and metabolic diseases (E) | 0 (0.0%) | 4 (100.0%) | 0 (0.0%) | 0 (0.0%) | 4 (0.6%) |
| Diseases of the genitourinary system (N) | 0 (0.0%) | 1 (100.0%) | 0 (0.0%) | 0 (0.0%) | 1 (0.1%) |
| Injury, poisoning and certain other consequences of external causes (S or T) | 0 (0.0%) | 1 (100.0%) | 0 (0.0%) | 0 (0.0%) | 1 (0.1%) |
| One reported as asymptomatic  Counts reflect the total number of individual adverse events (signs/symptoms) categorized by organ system and severity. A single call may include multiple adverse events. | | | | | |
